# Horizontal head-shaking test: pathophysiological mechanisms and clinical interpretation

**DOI:** 10.3389/fneur.2025.1735948

**Published:** 2026-01-07

**Authors:** Vincenzo Marcelli, Beatrice Giannoni, Giampiero Volpe, Michele Cavaliere, Edoardo Marcelli, Mario Faralli, Anna Rita Fetoni, Vito E. Pettorossi

**Affiliations:** 1 Department of Neuroscience, Reproductive Science and Dentistry, Section of Audiology, University of Naples “Federico II”, Naples, Italy; 2 Department of Neuroscience, Psychology, Drug’s Area and Child’s Health, University of Florence, Florence, Italy; 3 Department of Neurology, Ospedale San Luca di Vallo della Lucania, ASL, Salerno, Italy; 4 Faculty of Medicine and Surgery, University of Salerno, Salerno, Italy; 5 Department of ENT, University of Perugia, Perugia, Italy; 6 Department of Medicine and Surgery, University of Perugia, Perugia, Italy

**Keywords:** head shaking induced nystagmus, head shaking test, unsteadiness, velocity storage mechanism (VSM), vertigo

## Abstract

The horizontal head-shaking test (HST) is a simple, rapid, and non-invasive bedside maneuver that provides valuable insights into both peripheral and central vestibular function. Originally described in the late 19th century and standardized in the 1970s, the HST predominantly stimulates the lateral semicircular canals (LSCs) at high frequencies. Its diagnostic power lies in revealing dynamic asymmetries of the angular vestibulo-ocular reflex (aVOR) and uncovering central abnormalities involving the brainstem and, above all, cerebellum. The pathophysiological basis of post-head-shaking nystagmus (post-HSN) derives from Ewald’s second law, which explains the excitatory–inhibitory imbalance between the labyrinths, further processed through the velocity storage mechanism (VSM). The VSM extends and integrates canal signals, aligning them with gravity through the action of the cerebellar nodulus and ventral uvula. Its modulation determines the direction, duration, and morphology of post-HSN, accounting for central features such as perverted or minimal-stimulus responses. The VSM is furthermore crucial for higher-order functions such as motion perception, spatial orientation, and postural stability. Clinically, the HST contributes to discrimination between peripheral lesions—typically producing monophasic or biphasic horizontal post-HSN—and central disorders, which yield vertical or torsional (perverted) responses, or exaggerated responses due to cerebellar disinhibition. Thus, rather than an empirical test, the HST represents a neurophysiologically grounded tool that bridges bedside observation and the vestibular integration. When interpreted within a comprehensive clinical framework, it offers diagnostic, prognostic, and educational value, serving as a window into the physiology and pathology of vestibular–cerebellar networks.

## 1 Introduction and historical background

Neuro-otological research has long sought simple, reproducible, and sufficiently sensitive bedside clinical tests to detect subtle alterations in vestibular function. Among these, the HST remains one of the oldest yet most clinically relevant methods. Its value lies in the ability to stimulate the vestibular system at high frequencies, thereby complementing tests such as caloric stimulation, which are more sensitive to low-frequency dynamics. The phenomenon was first observed by Adler in 1897 (1) and Bárány in 1907 (2) and later systematically described and introduced into clinical practice by Vogel (3, 4). From the 1970s onwards, with standardized execution parameters and improved understanding of pathophysiology, the HST became established as a non-invasive bedside test with established diagnostic value. Seminal studies confirmed its ability to reveal unilateral or asymmetric bilateral dynamic dysfunction of the LSCs (5), as well as central alterations involving the medulla (6), pons (7), and especially the cerebellum (8). Accordingly, the HST provides clinically relevant information on both peripheral vestibular function and central vestibular pathways, including VSM. The VSM is crucial not only for reflexive oculomotor control but also for higher-order functions such as motion perception, spatial orientation, postural stability, compensatory adaptation and plasticity. The study of post-HSN therefore provides insights into the interaction between peripheral vestibular input and central processing.

## 2 Methodology of the horizontal head-shaking test (HST)

Accurate execution of the HST is essential for reliable interpretation. The standardized version of the HST involves several key steps, each grounded in specific physiological principles. The patient is seated under visual deprivation, achieved with Frenzel goggles or infrared video-oculoscopy, to prevent suppression of nystagmus by fixation. The head is pitched forward by approximately 20° in the pitch-down direction to align the LSCs more closely with the horizontal plane, thereby optimizing their bilateral stimulation (9). Although this maneuver reduces the vertical canals activation, some cross-stimulation remains unavoidable due to their non-orthogonal orientation to the horizontal plane. Experimental data indicate that the gain of the vertical canal aVOR is about 0.4 in the upright position and decreases to approximately 0.1 when the head is pitched forward by 30° (10). On this basis, a 20° forward tilt is widely considered the optimal compromise, maximizing LSC activation while minimizing vertical canal involvement (11). With the head properly positioned, the examiner firmly holds it and performs passive horizontal oscillations at a frequency of about 2 Hz, usually synchronized with a metronome or acoustic signal. Each excursion reaches about 45° to either side, with a total stimulation lasting approximately 15 s. The angular velocity should exceed 160°/s, which is considered sufficient to elicit post-HSN in the presence of vestibular asymmetry (11, 12). At the end of stimulation, the patient is asked to look straight ahead, while eye movements are observed for at least 20–30 s. Systematic documentation of post-HSN should include its direction, spatial plane, morphology (monophasic or biphasic), duration, and suppression with fixation. Under physiological conditions, the directional asymmetries generated by high-frequency sinusoidal stimulation are balanced between the two labyrinths, preventing the occurrence of nystagmus. The presence of post-HSN therefore indicates a functional asymmetry, whether of peripheral or central origin.

## 3 Pathophysiological mechanisms underlying the HST

### 3.1 Ewald’s second law

The fundamental physiological basis for post-HSN is Ewald’s second law, which describes the directional asymmetry of the aVOR in the LSCs: an ampullopetal flow (toward the utricle) produces a stronger excitatory response than an ampullofugal flow (away from the utricle), which exerts only a weaker inhibitory effect (11–13). Since the nonlinear inhibitory responses of the contralateral vestibular pathway quickly reach their saturation point, the aVOR becomes predominantly driven by excitatory input from the stimulated LSC (13), producing a net bias toward the side with higher neural activity (14). In cases of unilateral lesions, afferent responses from the intact labyrinth prevail over those of the impaired side, a disparity further accentuated by the nonlinear nature of inhibitory cutoff. Consequently, even under conditions of symmetric stimulation, repeated oscillations yield a sustained predominance of discharge from the intact labyrinth. This produces a persistent asymmetric input that drives the VSM, culminating in the generation of a post-HSN.

### 3.2 Functional role of the VSM: from mechanical to higher-order cortical processes

Mechanically, the VSM amplifies and prolongs semicircular canal signals, enhancing the aVOR during sustained or low-frequency head rotations (15–17) and during natural motion (18). Strongly influenced by gravity (19), VSM reorients toward the horizontal plane the aVOR, optokinetic responses (OKR), and optokinetic after-responses I and II (OKAN I and II) (20–25), supports cross-axis coupling and contributes to the perception of self-motion and spatial orientation (26–28). Thus, the VSM constitutes a bridge between reflexive eye movements and higher-order perceptual processes, contributing to self-motion perception, spatial orientation, and motion sickness susceptibility (28, 29). Furthermore, the VSM function is manifested clinically through tests such as the head-shaking paradigm, which underscores its pivotal role in reflexive and perceptual vestibular functions. This explains why patients with velocity storage dysfunctions often complain not only of oscillopsia or imbalance, but also of profound disturbances in spatial perception, motion sickness, and vertigo resistant to simple compensatory strategies.

### 3.3 Central nervous system and the VSM: the brainstem

The VSM is sustained by nucleus prepositus hypoglossi, neural populations located within the medial and superior vestibular nuclei, including vestibular-only (VO) neurons, and vestibular plus saccade (VPS) neurons (30–34), commissural interconnections between vestibular nuclei (31) and other structures, including the rostral interstitial nucleus of the medial longitudinal fasciculus (riMLF) and the interstitial nucleus of Cajal (INC) (35). Within this system, the principal target neurons are position-vestibular-pause (PVP) neurons and eye-head-velocity (EHV) neurons. These neuronal populations are essential for encoding and transmitting velocity-related information to ocular motor pathways (36–41); they are also crucial for integrating vestibular input in such a way as to sustain and precisely modulate the velocity signal underlying the aVOR. This integration is critical for ensuring the accuracy and stability of gaze during head movements. Visual input also significantly engages the VSM, especially during full-field optokinetic stimulation, by prolonging its activity as canal-derived signals decline (15, 16).

Additional brainstem regions also contribute to the spatial orientation of the VSM. In particular, the nucleus of Roller and the intercalated nucleus of Staderini (RSn), both connected with horizontal and vertical velocity-position neural integrators, participate in aligning ocular responses with the plane of vestibular stimulation (41, 42).

### 3.4 Central nervous system and the VSM: cerebellar nodulus and ventral uvula

The nodulus and ventral uvula (NvU) play a central role in inhibitory control of VSM, finely regulating its time constant and spatial orientation, aligning its dynamics with gravity (26, 27, 43). Purkinje cells within the NvU receive direct utricular afferent inputs (27, 44) and project exclusively to the ipsilateral vestibular complex (45, 46). Functionally, NvU neurons exert dual regulatory functions. First, they integrate signals from otolith organs and semicircular canals to adjust orientation dynamics, thereby shaping three-dimensional spatial orientation (26, 47, 48). Second, through reciprocal connections with the superior and medial vestibular nuclei and the inferior olive, they process vestibular and optokinetic inputs in a conserved manner across species (27, 45, 48, 49). A paradigmatic example of this regulatory function is tilt suppression (also referred to as “dumping”), first described by Benson et al. in 1966 (50). After constant-velocity rotation around the earth-vertical axis, pitching the head creates a mismatch between graviceptive and rotational cues, generating inappropriate nystagmus. NvU-mediated compensation restores alignment by shifting the axis of post-rotatory nystagmus toward the gravitational vertical, reducing the aVOR time constant from approximately 20 s to 7 s, and attenuating nystagmus intensity (43, 51). Due to its simplicity and reproducibility, tilt suppression has become a reliable clinical test for assessing vestibulo–cerebellar function, particularly the integrity of the nodulus and uvula (52, 53). From a mechanistic point of view, NvU neurons not only discharge stored velocity signals but also dynamically adjust the VSM time constant when eye velocity exceeds surrounding motion (54) and finely tune VSM cross-coupling parameters to ensure eye velocity alignment with gravito-inertial acceleration (26, 27, 43, 46, 55–58). Functionally, lateral NvU regions predominantly regulate horizontal dynamics, whereas central regions modulate vertical and torsional components (26, 27, 32, 44). At the cellular level, this inhibitory control is mediated through GABA_B_ receptors (43, 59, 60). Notably, in cats this inhibition is strictly lateralized — either ipsilateral or contralateral, but never bilateral (60). The pathophysiological relevance of NvU function is highlighted by lesions that typically give rise to periodic alternating nystagmus (PAN), characterized by an abnormally prolonged aVOR time constant (44). Clinically, PAN can be effectively controlled in about two-thirds of patients with administration of the GABA_B_ receptor agonist baclofen (61), underscoring the therapeutic significance of this mechanism. Moreover, NvU lesions generate aVOR time constant alterations, absence of optokinetic after-nystagmus, and misoriented or prolonged nystagmus (20, 44).

### 3.5 Which vestibular stimulus activates the VSM?

Recent physiological research has identified two distinct vestibular circuitries activated by different stimuli. According with this study, skull vibration, head impulses, and vestibular-evoked myogenic potentials (VEMPs) are guaranteed mainly by type I vestibular afferents with irregular discharge, which are highly sensitive to phasic and transient stimuli (62–72). Such a “direct” pathway bypasses the VSM, generates very rapid short-latency compensatory eye movements, and its effects decay quickly once the stimulus ends (62, 66, 68, 73). This pathway does not contribute significantly to the sustained sense of self-stability that patients often report as impaired. In contrast, stimuli such as caloric irrigation, low-frequency rotation, optokinetic, or head-shaking responses (64, 74) are mainly driven by type II vestibular afferents with regular discharge, which respond preferentially to these tonic and sustained stimuli. This “indirect” pathway integrates both vestibular and optokinetic inputs through the VSM, which, as previously underlined, prolongs semicircular canal responses and represents the core of the VSM (16, 18).

Notably, whereas vibration almost exclusively stimulates irregular afferents, during head shaking both regular and irregular afferents are activated (74–77). Supporting this, baclofen, known to inhibit the VSM, markedly reduces post-HSN but has little effect on skull vibration-induced nystagmus (SVIN, 61). Taken together, these findings confirm that transient responses mediated by type I afferents provide rapid motor compensation but do not engage the VSM, whereas type II responses mediated by regular afferents are crucial for activating the VSM and sustaining self-motion perception and spatial stability. Consequently, stimuli such as the HST specifically probe the VSM and are particularly sensitive to asymmetries in velocity storage function, which are thought to underlie much of the subjective sense of vestibular instability.

### 3.6 VSM and the head-shaking test

The HST offers a direct clinical window on velocity storage function. By transiently amplifying canal signals and extending their dynamics, velocity storage underlies the post-HSN. In unilateral vestibular lesions, asymmetry within the velocity storage generates a transient bias in slow-phase velocity. Importantly, because velocity storage is gravity-referenced, head-shaking responses also reveal the interaction between canal signals and otolithic input, linking the HST not only to canal asymmetry but also to the central mechanisms that align motion perception with the Gravito-Inertial Acceleration (GIA).

### 3.7 Post-head-shaking nystagmus (post-HSN)

Post-HSN is considered clinically significant when at least five consecutive beats are observed after cessation of head oscillation (78).

At the bedside, both qualitative and quantitative parameters should be assessed. Qualitative aspects include the morphology of the response (monophasic vs. biphasic), its direction, and the spatial plane of the nystagmus. Quantitative measures primarily refer to duration, amplitude/frequency (intensity), and consistency, the latter reflecting the variability of the response across different stimulus intensities and head-pitch positions.

### 3.8 HST in peripheral lesions: pathophysiology and clinical interpretation

Under normal conditions, when the dynamic gain of the LSCs aVOR is symmetrical, the transient directional asymmetries produced by sinusoidal head movements are balanced, and no net input reaches the VSM. In contrast, unilateral LSC or bilateral asymmetric LSCs dynamic gain abnormality amplifies these asymmetries, leading to directionally biased responses. In the absence of spontaneous nystagmus, this imbalance generates a predominantly horizontal post-HSN, typically beating toward the functionally dominant side, consistent with a dynamic uncompensated high-frequency vestibulopathy: the “paretic” post-HSN (16, 79, 80).

When spontaneous nystagmus is present, several scenarios may arise:

- Exacerbation of spontaneous nystagmus, suggesting coexistence of aVOR dynamic gain asymmetry with an underlying static imbalance.
- No change in spontaneous nystagmus, which may reflect acute-phase of partial or complete peripheral vestibular lesion, where the VSM is functionally deactivated to minimize static and dynamic symptoms; this is evidenced by a reduction of the dominant time constant to ~5 s, consistent with the cupula-endolymph system (79–81).
- Reversal of spontaneous nystagmus direction, traditionally considered a central sign such as in lateral medullary infarction (82), but also explainable by peripheral mechanisms:

- In “recovery” nystagmus, directed toward the lesioned side, whereas post-HSN beats contralesionally, reflecting static recovery of the damaged labyrinth but persistent dynamic dominance of the healthy side;
- In “irritative” nystagmus, often seen in advanced Ménière’s disease (MD), where utriculopetal endolymphatic pressure excites the ampullary crest, producing ipsilesional nystagmus, while mechanical compression prevents physiological responses to head-shaking, thereby inducing contralesional post-HSN (83). In such cases, vHIT confirms hypofunction of the “irritated” labyrinth (see video material, Supplementary Video 1. Irritative Ny in right MD).

Importantly, if HST amplifies a response toward the affected side rather than inverting it, the nystagmus should be reclassified as recovery rather than irritative. In summary, in the absence of additional central signs, we suggest that a reversal of spontaneous nystagmus direction during HST alone should not be considered evidence of a central lesion.

Finally, although we consider this a rare occurrence, it is worth recalling that a severe bilateral peripheral deficit of the posterior semicircular canals (PSCs) may give rise to a perverted HSN, as we have recently reported (84).

### 3.9 Monophasic and biphasic post-HSN: clinical patterns and interpretative hypotheses

Post-HSN may present as monophasic or biphasic. Monophasic responses consist of nystagmus consistently beating in one direction. “Paretic” monophasic nystagmus beats toward the dominant side; “recovery” monophasic nystagmus beats toward the hypofunctioning side which, in our experience, is relatively uncommon. It likely occurs within a narrow temporal window during the dynamic and high-frequency recovery phase of the previously impaired vestibular hemisystem, which may transiently regain dominance over the contralateral side, still inhibited by cerebellar modulation mechanisms designed to reduce static and dynamic asymmetry.

Biphasic responses begin with a generally high-intensity nystagmus in one direction, followed by spontaneous reversal. This phenomenon was first described by Kamei et al. (85) and later confirmed by Takahashi (86), who hypothesized that in cases of unilateral peripheral vestibular hypofunction the first phase represents a paretic nystagmus, whereas the second phase reflects a recovery response, indicating that the lesioned side has begun to regain function. Other authors have linked it to short-term adaptation of peripheral nerves (5) or to shortened time constants of VSM and gaze-holding integrators (87). We assume that second phase results from short-term adaptive mechanisms within both peripheral and central vestibular structures, triggered when the first phase is sufficiently intense to induce refractoriness of receptors or functional exhaustion of vestibular nuclei, allowing transient contralateral dominance (even in presence of hypoactive or inactive labyrinth). This requirement of a strong initial phase has been experimentally confirmed (78, 88). In practice, monophasic responses likely indicate milder asymmetry, while biphasic patterns reflect more severe or unstable dysfunction. Large spontaneous nystagmus may obscure this reversal, presenting only as transient attenuation rather than clear inversion.

### 3.10 Modulation by head pitch

Forward flexion of the head normally reduces post-HSN intensity, reflecting intact modulation of the VSM by cNU and utricular inputs (89). Absence of this suppression is a strong indicator of cNU dysfunction (43, 52, 53, 89).

### 3.11 HST in central lesions: pathophysiology and clinical interpretation

HST may generate distinctive responses in central vestibular disorders (90, 91). Five clinically relevant patterns are recognized: perverted post-HSN, disinhibited post-HSN and minimal-stimulus post-HSN, no response to tilt suppression test, no change of spontaneous nystagmus.

- Perverted post-HSN is defined as nystagmus emerging in a plane different from the head-shaking stimulus. Manifestations include:

- Appearance of vertical (upbeat or downbeat) or torsional (clockwise or counterclockwise) nystagmus predominating over horizontal components (82).
- Change in the orientation of the plane of a spontaneous horizontal nystagmus.
- Marked increase of a pre-existing downbeat nystagmus (82).

Lesions underlying this pattern include Flocculus–Paraflocculus Complex, which exert inhibitory control over floccular target neurons (FTNs) involved in the anterior semicircular canal (ASC) aVOR pathway, with a lesser influence on the PSCs pathway (92–94). Lesions in this area disinhibit ASCs projections and induce downbeat post-HSN (10, 92–94). Cerebellar Nodulus and Uvula (cNU) are critical for maintaining spatial orientation of the aVOR and their lesions causes cross-coupling between horizontal and vertical/torsional pathways, generating perverted responses (26, 27, 44, 94). Neural Integrator Coupling, where abnormal interaction between horizontal and vertical/torsional integrators (nucleus prepositus hypoglossi, interstitial nucleus of Cajal, RSn) produces inappropriate vertical outputs, sometimes due to ephaptic transmission in demyelinating disease (6, 15, 40–42).

- Disinhibited post-HSN (d-post-HSN) and minimal-stimulus post-HSN (ms-post-HSN), are, respectively, defined as an extremely intense post-HSN or as a post-HSN generated by only a few cycles of low-velocity head rotations insufficient to produce a significant imbalance and activate the VSM under normal conditions. This finding, regardless of the nystagmus plane, is highly suggestive of cNU lesions, which normally suppresses subthreshold vestibular inputs. Loss of this inhibitory control permits abnormal engagement of the VSM and exaggerated responses (43). Notably, ms-post-HSN may also be purely horizontal. Asymmetric disinhibition of the VSM from unilateral or bilateral or unequal involvement cNU involvement can indeed mimic peripheral post-HSN, as reported in lateral medullary infarction (60) and unilateral cerebellar infarction (95).
- No response to tilt suppression test. The failure to reduce the post-HSN intensity with the forward flexion of the head normally must raise suspicion of a lesion of cNU (43, 51–53).
- No change in spontaneous nystagmus. The peripheral stimulus does modify the spontaneous nystagmus, unlike in labyrinthine deficits where modulation is expected.

In order to provide a practical clinical overview of what has been discussed so far regarding the pathophysiology of HST on the horizontal plane, we include as an integral part of our work a video atlas illustrating cases of patients who showed the findings described in detail and their underlying pathology. We believe that this can be of immediate help and useful comparison for those who interact with vertigo patients in all clinical settings, from the first point of contact doctor to the third-level specialist.

## 4 Conclusion

Despite its simplicity, the HST provides rich information on peripheral and central vestibular function. Since its introduction into clinical neurotology, substantial progress has been made not only in refining the interpretation of its results but also in elucidating the pathophysiological mechanisms underlying the different patterns of post-HSN. This has allowed the HST to evolve from a purely empirical tool into a test with a solid neurophysiological basis, capable of offering valuable insights into the dynamics of the aVOR, the VSM, and their modulation by cerebellar and brainstem structures.

Despite its apparent simplicity, the HST requires careful observation and interpretation. Clinical practice demonstrates that the variety of possible responses, ranging from monophasic to biphasic, from paretic to recovery nystagmus, as well as the more complex perverted or minimal-stimulus forms, demands both theoretical knowledge and practical experience. In fact, not all clinicians have the opportunity to directly encounter and analyze the full spectrum of vestibular and central pathologies associated with these responses. For this reason, correlating physiopathological explanations with real clinical cases, as we have attempted in this work, is of particular educational and clinical value.

A thorough understanding of HST findings has several implications. In the acute care setting, it can support emergency physicians in rapidly distinguishing between peripheral and central vestibular disorders, thereby guiding decisions regarding immediate referral or advanced imaging. For specialists, such as otoneurologists and neurotologists, the HST remains an essential tool to complement vestibular function testing, helping to orient the diagnostic pathway, refine differential diagnosis, and select appropriate therapeutic strategies. Moreover, when interpreted within a broader clinical and instrumental context, the HST can provide prognostic indications, for example by distinguishing between phases of decompensation, recovery, or central maladaptation.

Ultimately, the clinical value of the HST lies in its ability to probe vestibular integration mechanisms through a simple bedside maneuver. Its clinical utility is therefore maximized when the test is not considered in isolation but rather interpreted in the light of a deep understanding of the mechanisms that generate the observed responses. In this way, the HST continues to serve as both a practical diagnostic maneuver and a powerful teaching tool, bridging the gap between clinical phenomenology and the underlying neurophysiology of balance.

## Data Availability

The original contributions presented in the study are included in the article/Supplementary material, further inquiries can be directed to the corresponding author.

## Supplementary material

SUPPLEMENTARY VIDEO 1 HSN. First and second phase.

SUPPLEMENTARY VIDEO 2 HST. Increase of spontaneous nystagmus.

SUPPLEMENTARY VIDEO 3 Right MD. Irritative right beating nystagmus and left beating nystagmus post-HSN.

SUPPLEMENTARY VIDEO 4 Perverted post-HSN due to severe bilateral posterior semicircular canals deficit.

SUPPLEMENTARY VIDEO 5 Disinhibited post-HSN due to vascular lesion of nodulus and uvula.

SUPPLEMENTARY VIDEO 6 Perverted post-HSN due to neoplastic lesion of nodulus and uvula.

SUPPLEMENTARY VIDEO 7 HST. Increase of spontaneous DBN in Fazekas 5 patients.
